# Supplementary material for: Evaluation of a ddPCR Commercial Assay for the Absolute Quantification of the Monkeypox Virus West Africa in Clinical Samples
Source: Diagnostics (Basel). 2023 Apr 4;13(7):1349. doi: 10.3390/diagnostics13071349 (PMC10093040; doi:10.3390/diagnostics13071349)
Supplement: Supplementary file 1 [file diagnostics-13-01349-s001.zip › diagnostics-2298542-supplementary/Supplementary information.pdf]

Figure S1. Sequence of the synthetic Gblock DNA.

Synthetic DNA Sequence for Positive Controls

Gblock Sequence:

CCCATGAGACATACAAAAAGGTAATGCCGCCTCGCTAGGTGAGCTACAGCTCGATT
GTCACGTTAAGCTGGCCAGTTCAAATCCCTGTTGCATTAATTTCACCAGTAATGAGT
CTTTTTCGATACAGGTTAATTTCCACATCGATATAGTTAAATGTATTGCTGGTTACGA
CGGGTTCGCATTTATCTGTGGAAGAGACGGTGTGTGACCTGGCTGTAGCTTAGGA
GTAGCATGTTCTTTACGATCATAGTTCATTCATGAAACTATTTTATTCATCTCTCGGT
GAAGCTTCAGAGAACTTTATTAGGTATGTTTACTTAACAAAAGAGTGCATTGGGGGT
GATGAAGCCTAGTCAAATTCACAGAAAGCTAAGGAGGTCTCGACTATACGCCCGTT
TTCGGATC

Legend of excel files

Supplementary material Table S1. ddPCR data sets of LoD experiments.

Supplementary material Table S2. ddPCR data sets in the clinical samples.
